# Cemented vs uncemented megaprostheses in proximal femur metastases: a multicentric comparative study

**DOI:** 10.1186/s12891-022-05726-7

**Published:** 2022-09-06

**Authors:** Maria Serena Oliva, Francesco Muratori, Raffaele Vitiello, Antonio Ziranu, Lorenzo Foschi, Giuseppe Rovere, Cesare Meschini, Domenico Andrea Campanacci, Giulio Maccauro

**Affiliations:** 1 Fondazione Policlinico Universitario Agostino Gemelli – IRCCS, Largo Agostino Gemelli 8, 00168 Rome, Italy; 2 Università Cattolica Del Sacro Cuore, Rome, Italy; 3 Ortopedia Oncologica e Ricostruttiva Azienda Ospedaliera Universitaria Careggi, Florence, Italy

**Keywords:** Metastases, Proximal femur, Megaprostheses

## Abstract

**Background:**

Hip megaprostheses are a long known reconstructive method in the treatment of proximal femur metastases. The use of cemented or uncemented stems is still matter of debate. The aim of this study to compare cemented and uncemented megaprostheses on functional outcomes and complications, in order to establish the role of cementation.

**Methods:**

We retrospectively analysed 51 metastatic patients with proximal femur metastases treated with endoprosthetic reconstruction by megaprostheses, 25 with cementless stems and 26 with cemented ones with different megaprosthetic implants. The primary endpoint was MSTS score, and the secondary endpoint was to state the incidence of surgical and clinical complications in the two groups. An un-paired T test was used to compare anthropometric, anamnestic data, and MSTS. Chi-square test was performed for evaluation of complication in the two group. Multiple linear regression was used to match the functional outcomes and complications’ incidence in the population study. Logistic regression was performed to analyse the odds ratio of different parameters and their role in the incidence of complications.

**Results:**

The mean follow-up was 50.1 months (+ 12.5). In thirty case right side was involved. No statistical differences were noticed between Group A and B regard the age, gender, active fracture/impending fracture. Comparing the MSTS results within the two groups at last follow-up, the score cemented group was higher than cementless one (17.9 + 7.8 vs 24.2 + 5.3; statistical significance *p* = 0.001). Regarding surgical complications a logistic regression was performed to analyse the odds ratio of age, cementation and length of resection; cementation confirm and odds ratio of 11 times in the incidence of surgical complications.

**Conclusions:**

Cementation seems to be more liable to complications onset, while improves functional score in metastatic patients compared to uncemented megaprostheses. More studies have to be conducted in order to create a protocol and establish criteria to use cemented or uncemented stems in a frail population like metastatic patients.

**Supplementary Information:**

The online version contains supplementary material available at 10.1186/s12891-022-05726-7.

## Background

Proximal femur is one of the most common bony location of metastatic disease [1, 2]. Proximal femur metastases develop in about 10% patients with primary malignant tumor [3, 4]. Impending or pathological fractures have to be surgically treated ensuring local control of the disease, pain control and good functional and clinical outcomes [5, 6].

The use of hip megaprostheses in the management of hip metastases has been largely known from years now [7]. Megaprostheses in solitary skeletal-related events can guarantee adequate surgical margins, pain relief and a stable joint situation allowing fast patient mobilisation after surgery [8].

Megaprosthetic implants can be either cemented or uncemented and implant-related complications [9], such as dislocation, infection and aseptic loosening have been described for both kinds of implant [10, 11].

Some authors prefer cementation because of lower rates of revision for loosening and no need of osteointegration for total weight bearing, so this condition allows patients to start chemotherapy as soon as possible [9, 12, 13].

On the other side cementation increases surgical time length and consequently infection risk [14], and in literature some authors described “bone cement implantation syndrome” with higher risk of developing intra-operative death and higher risk of pulmonary embolism [15, 16]. Lastly the presence of a cemented megaprosthesis could aggravate subsequent revision surgeries.

Some authors recommend cementation to be performed in patients who need postoperative radiotherapy and/or with additional metastases, while in all other cases cementation is not needed [17].

In literature cementless stem endoprostheses reported higher implant survival and infection survival compared to cemented ones, while the aseptic loosening rates were similar in the two groups [9].

Whether some authors compared cemented and cementless megaprosthetic implants about survival and infection rates others compared nail fixation with endoprosthetic reconstruction and their functional outcomes [3, 9]. The use of cemented or cementless stem prostheses is still matter of debate.

The aim of this multicentric retrospective study is to understand the role of cementation in patients affected by proximal femur metastasis treated with modular endoprostheses and to compare complication rates and functional outcomes.

## Methods

Metastatic patients treated with proximal femur resection and modular endoprosthetic reconstruction in two major oncologic orthopaedic hospitals were retrospectively analysed.

**Fig. 1:**
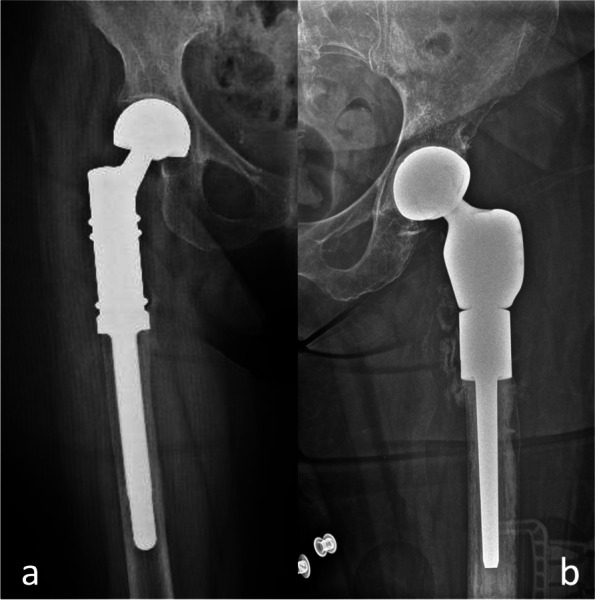
(**a**) Uncemented megaprosthesis, (**b**) Cemented megaprosthesis

Group A used cementless megaprostheses (Fig. 1a), while Group B used cemented megaprostheses (Fig. 1b).

Inclusion criteria were: metastatic disease in patients > 18 years old with proximal femur (from greater trochanter to sub-trochanteric area) resection and endoprosthetic reconstruction.

Exclusion criteria were: patients with follow up less than 6 months, patients with primary bone tumour of the proximal femur, distal or total femur resection/reconstruction.

### Surgical technique

All patients underwent hip endoprosthetic reconstruction by megaprosthesis.

In group A all the procedures were performed by fellowship-trained in oncological surgery orthopaedic surgeons.

General anesthesia was performed in all cases. All patients received Cephazoline 2 g i.v. as antibiotic prophylaxis before surgery, if not contraindicated [18]. A urinary catheter was placed in all patients and removed within 72 hours after the surgery.

Patients were placed in lateral decubitus position. A lateral approach was used. After bone exposure, an en bloc resection was performed, cementless silver-coated megaprosthesis was implanted according to the manufacturer technique (Mutars, Implantcast) [19]. The surgery was completed by myodesis through the Trevira Tube© (Implantcast; GmbH, Buxtehude, Germany). One intra-articular closed-suction drainage was placed and then removed 48 hours after surgery.

In group B all the procedures were performed by fellowship-trained in oncological surgery orthopaedic surgeons.

General anesthesia was performed in all cases. All patients received antibiotic prophylaxis before surgery according to hospital’s protocol, if not contraindicated.

Patients were placed in lateral decubitus position. A lateral approach was used. After bone exposure, an en block resection was performed, cemented endoprosthesis was implanted according to the manufacturer technique (Megasystem C Link) [20]. The surgery was completed by myodesis through pre-existing prosthetic holes. One intra-articular closed-suction drainage was placed and then removed 48 hours after surgery.

All patients followed the same post-operative rehabilitation protocol: at 48 hours after surgery patients were seated with their feet out of bed; at 72 hours, they were allowed to progressive weight bearing with walker frames.

### Clinical evaluation

Anthropometric and anamnestic data, primitive tumor, presence of impending or pathological fracture, surgery time were collected.

All patients restarted oncological cares 30 days after surgery according to oncological protocols.

Complications were divided into two groups: surgical and clinical ones. In the surgical complications group were included: wound dehiscence, deep infection, dislocation, aseptic loosening, revision rate.

Wound dehiscence or surgical site infection was defined as a delayed of normal healing of the surgical wound with presence of redness, edema and secretion in absence of deep tissue involvement or general symptoms [21].

In the clinical complication group pneumonia, pulmonary embolism, urinary tract infection, post-operative anemia, decubitus, development of deep vein thrombosis were included.

Self-assessed questionnaires were administered to all patients at the clinic at 6 months follow-up. Muscoloskeletal Tumor Society scoring system (MSTS) the scale was given to all patients to evaluate their residual post-operative function [22]. The scale evaluates 6 parameters (Pain, Function, emotional, supports, walking, gait) in a scale from 0 to 5 (0 minimum score, 5 maximum).

### Radiological assessment

Fractures were diagnosed through a standard X- ray series in each case (AP and LL). Impending fractures were identified with standard X-ray series and in peculiar cases with the aid of TC scan and Mirels’score was used to stratify the fracture risk [23]. For a Mirels’score over 8 points surgical indication was confirmed. Affiliation was used to identify Femur MRI with radiocontrast has been used to assess the bone resection length.

An X-ray (AP and LL) was required at first, third and sixth postoperative month and then every year.

The patients underwent to a total body CT by oncologists’ decision and came to the orthopaedic clinic to evaluate the disease’s progression.

### Statistical analysis

GraphPad QuickCalcs (GraphPad Software, San Diego) was used for data analysis. The data were reported as mean and standard deviation (+ SD).

The asymmetry was calculated to evaluate the normality of the different parameters.

An un-paired T test was used to compare anthropometric, anamnestic data, and MSTS. Chi-square test was performed for evaluation of complication in the two group. Multiple linear regression was used to match the functional outcomes and complications’ incidence in the population study. Logistic regression was performed to analyze the odds ratio of different parameters in the incidence of complication.

Significance was set for *p* < 0.05.

## Results

Fifty-one patients were considered eligible according to inclusion and exclusion criteria and were finally included in the study.

Twenty-five patients were assigned to Group A (uncemented) and twenty-six in Group B (cemented).

The primary outcome analyzed was the clinical outcomes at last follow-up (MSTS score). The secondary outcome was incidence of complications.

Breast cancer was the most common primary tumor (35%), followed by kidney (13%), lymphoma (13%), lung (9%), prostate (9%), myeloma (7%) and other (10%).

In Group B, preventive surgery was more frequent than Group A, but without statistical difference (44% vs 57%; *p* = 0.3).

There were 18 male and 33 females, the mean age was 64,8 years old (+ 2.4). The mean follow-up was 50,1 months (+ 12.5). In thirty case right side was involved. No statistical differences were noticed between Group A and B regard the age, gender, active fracture/impending fracture (Table 1).

**Table 1:** Baseline characteristics

	**Total**	**Cementless**	**Cemented**	***P***
**Patients**	51	25	26	
**Sex**	18 M | 33 F	9 M | 16 F	9 M | 17 F	
**Age (years)**	64.8 ± 2.4	67.5 ± 10	62 ± 13	0.1
**Active fracture**	25	14	11	0.3
**Impending fracture**	26	11	15	0.3
**Follow-up (months)**	50.1 ± 12.5	39 ± 33	61 ± 51	0.1
**Duration time of surgery (hours)**	2.6 ± 0.2	2.7 ± 0.6	2.4 ± 0.9	0.7
**Resection length (cm)**	12.6 ± 0.5	11.8 ± 3.3	13.5 ± 3.8	0.08
**MSTS**	21.1 ± 1.8	17.9 ± 7.8	24.2 ± 5.2	0.001*

In brackets measurement unit; Data were reported as absolute value ± SD. * underline statistical significance

No difference was noticed among surgery duration time (Group A 2.6 + 0.6 hours vs Group B 2.4 + 0.9 hours; *p* = 0.7) or resection length (Group A 11.7 + 3 cm vs Group B 13.5 + 3.8 cm; *p* = 0.08) (Table 1).

Comparing the MSTS results within the two groups at last follow-up, the score in Group B was higher than Group A (17.9 + 7.8 vs 24.2 + 5.3; *p* = 0.001).

Globally fourteen complications were recorded.

In Group A, two urinary tract infection (2/25 patients; 8%), one pneumonia (1/25 patients; 4%) and one dislocation (1/25 patients; 4%) were recorded. In Group B, one deep vein thrombosis (1/26 patients; 3%), three wound dehiscence (3/26 patients; 11%), one nerve paresis (1/26 patients; 3%), one aseptic loosening (1/26 patients; 3%) and four dislocations (4/26 patients; 15%) were recorded (Table 2).

**Table 2:** Complications

	**Total**	**Cementless**	**Cemented**	***P***
**Patients**	51	25	26	
**Surgical complications**	10 (19%)	1 (4%)	9 (34%)	0.005
**Dislocations**	5 (9%)	1 (4%)	4 (15%)	0.15
**Wound dehiscence**	3 (5%)	0	3 (11%)	
**Nerve palsy**	1 (1%)	0	1 (3%)	
**Aseptic loosening**	1 (1%)	0	1 (3%)	
**Clinical complications**	4 (7%)	3 (12%)	1 (3%)	0.2
**Urinary tract infections**	2 (3%)	2 (8%)	0	
**Pneumonia**	1 (1%)	1 (4%)	0	
**Deep vein thrombosis**	1 (1%)	0	1 (3%)	

In brackets measurement unit; Data were reported as absolute value and percentage. * Underline statistical significance

Statistical difference was not noticed between the two groups regarding dislocation (1 vs 4; *p* = 0.15) but grouping the surgical complication (1 vs 9; *p* = 0.005) but not for clinical complication (3 vs 1; *p* = 0.2).

All patients affected by infections were successfully treated by antimicrobial oral therapy with complete resolution, with no need of further surgery. Dislocation was treated by open reduction and implantation of acetabular bis mobility cup. Nerve injury was treated by orthopedic brace. Aseptic loosening needed an implant revision. Deep vein thrombosis was treated by anticoagulant therapy and evaluated at follow-up with a series of ultrasounds and doppler.

A multivariate model was performed matching gender, age, cementation and resection length regarding MSTS; only cementation confirms his statistical significance (*p* = 0.002).

Regarding surgical complication a logistic regression was performed to analyze the odds ratio of age, cementation and length of resection; cementation confirm and odds ratio of 11 times in the incidence of surgical complications (Table 3).

**Table 3:** Logistic regression of surgical complication

**Descriptor**	**Odds ratio**	**95% Confidence interval**	***P***
**Age**	1	0.9–1.1	0.3
**Cementation**	11	1.1–103.7	0.03*
**Resection length**	1	0.8–1.3	0.4

* underline statistical significance

## Discussion

Proximal femur is one of the most common bone metastases localization [3]. Often metastases develop subtly and give scarce signs of their presence and diagnosis is made when symptoms of pathological fracture are evident [24–26].

Endoprosthetic reconstruction is a widely used reconstructive technique in the treatment of proximal femur metastases [27]. This kind of surgery has implant-related complications, due to the large bone resections and soft tissue excision often needed to guarantee oncological radicality [10, 27–29].

The use of cemented or uncemented stems following megaprosthetic hip reconstruction is still matter of debate. Cemented stems surely guarantee immediate weight-bearing because the stem does not need osteointegration and cementation itself guarantees a better grip [9].

Bischel et al. in their paper demonstrate the validity and safety of uncemented stem positioning in metastatic femur [17, 30], while Griffin et al. stated how cementless fixation may be advantageous because of bone ingrowth that may lead to very low aseptic loosening rate [28].

Moreover cementation needs longer surgical time that increased infection risk and in literature “bone cement implantation syndrome” was described, characterized by hypoxia, hypotension, cardiovascular collapse and an increased risk of pulmonary embolism [15, 16]. Lastly the presence of a cemented megaprosthesis could aggravate subsequent revision surgery.

We performed this retrospective study of metastatic patients treated with endoprosthetic reconstruction to evaluate the role of cementation on functionality and surgical complications onset.

Our result show how stem cementation positively affects patients’ functionality and restoring of daily activities compared to uncemented stems (24.2 ± 5.2 of the cemented group vs 17.9 ± 7.8 of the uncemented one), while increases the surgical complications if compared to uncemented stems (9 vs 1 in the uncemented group).

The multivariate analysis in our study showed that cemented megaprostheses have functional better outcomes compared to the cementless. This result is in line with literature findings [30, 31], cementation allows immediate weight bearing and faster restoring of function. Angelini et al. showed in their study about cemented proximal femur replacements (PFR) that the mean MSTS score in the post-operative period was 22.4 points, similar to our study findings in cemented group.

Ferrara et al. showed how also in uncemented PFR the MSTS improves at 2 months post-operative controls and that these patients can improve their gait modalities and functional daily life outcomes until 3 months from surgery [32–34].

Another finding of this study is about complication rate. In the multivariate analysis cementation compared to age, sex, resection length appears to be the only variable influencing complications.

Dislocations represent another well know complication of hip megaprostheses in literature [35–38], we found it in this study. Prostheses dislocation are more frequent in cemented group compared to uncemented group. This can be related to the design of the prostheses, different in the two groups and to the presence of Trevira tube (only in cementless group). Literature data suggest that dislocation rate is 3–22% in this kind of surgery [9, 35–37, 39]. D’Adamio et al. in their study confirm the in vitro safety and efficacy, in terms of newly formed cells extension and adhesion pattern, of using an attachment tube made from Trevira fibers surrounding an oncological megaprosthesis [40, 41]. This factor could have improved the most anatomical reinsertion of remaining soft tissue following resection.

Furthermore, if Trevira Tube according to some authors can assure soft tissue adhesion [41], can on the other the other side impede a close reduction [17]. This means that dislocation in cases where Trevira tube has been used need an open reduction, and consequently a reintervention.

Also, infection rate is higher in cemented group compared to uncemented group. This result is in line with literature results [42]. Piccioli et al. involved 30 patients in their study about lower metastasis, but just 11 of them were treated for PFR [33]; in this case complications presented in 30% of the population with an infection rate of 16.7%. It is not specified if the proximal femurs were treated for primary tumor or metastatic disease and if complications occurred in PFR, due to the variously treated patients (total femur, proximal tibia, proximal humerus). Also, authors that used both techniques, cemented and cementless megaprostheses, presented delayed wound healing, infection and aseptic loosening. Pala is the only author that compares cemented and uncemented megaprostheses and reports that infections and aseptic loosening are higher in cemented mega prosthesis replacements [9, 43].

Besides Donati et al. reported in their study that silver coating of megaprostheses can decrease infection rate [44, 45], due to the release of silver ions, which produces a zone of growth inhibition for bacterial activities, and confirmed the protective role of silver coating in the first 6 months after surgery [10, 44, 45]. Cementless group in our study actually has been treated with silver-coated megaprostheses, and this can be considered a protective factor for the development of infections, which did develop as complication in cemented group.

In our study we reported one case of aseptic loosening in one patient with cemented stem. This result is confirmed by literature trend, in fact cementation seems to be a more frequent cause of aseptic loosening [46]**.**

This study has some limitations, first its retrospective design and the examined population. Patients with femoral metastases in fact have a variable life expectancy [47, 48], that varies from weeks to years, and many patients die within 2 years after surgery [38, 49, 50]. Number and location of metastases, primitive tumor, presence of pathologic or impending fracture, age, can affect the prognosis [51–53]. Of course, the presence of pre-existing pathologies must be considered in the evolution of the prognosis, but these data are not disposable. All these data generate very large confidence intervals in an already small population. In light of these limitations, even in the presence of significant data, it is desirable in the future to prepare studies with the recruitment of more patients, so as to be able to carry out broader and more rigorous multivariate analyzes.

Another limit of the study is represented by the different prostheses used for limb reconstruction. Silver coating, as already said, literature agree to be a protection against early infection [33, 44, 54, 55]. Secondly, the presence of a different design to guarantee soft tissue adhesion: while Mutars (Implantcast) provides Trevira Tube to reattach tendons and muscular insertions, Megasystem C (LINK) provides pre-existing holes on the prostheses, through which tendons and muscles must be assured. This difference in design may play a role in dislocation onset.

Even if megaprostheses tend to dislocation because of the resection of muscular insertion and often the lack of capsular stability, there are also patient-related factors that can improve the onset of this condition, such as the presence of a coxa plana or the large soft tissue resection, which is not quantifiable. Both these factors have not been considered and/or reported for patients in cemented and cementless groups, and this represents another limit for the present study.

Despite the limitations, our study presents different strong points: first the considered population is screened for only proximal femur metastasis, differently from other studies in which also primary tumours or other bone locations were considered [9, 44]. All the patients underwent endoprosthetic replacement and reached a long-term follow-up of about 50 months. Only few studies in literature analysed functional outcomes like MSTS in proximal femur megaprosthesis replacement [3].

## Conclusions

Stem cementation in hip endoprosthetic reconstruction by megaprostheses in metastatic patients is still matter of debate. Whether cementation guarantees better functional results, it seems to be associated to higher complication rates. Future studies based on the use of the same kind of endoprosthetic implant have to be performed to confirm our results and to propose a universal guideline about the use of cemented or uncemented stems in tumoral endoprosthetic reconstructions.

### Supplementary Information

**Additional file 1: Supplementary Fig. 1.** Supplementary data reported with confidence interval. Follow-up reported in months, age in years old. Duration time of surgery in minutes. Resection length in cm. * for statistical significance of *p* = 0.001.

**Additional file 2: Supplementary Fig. 2.** Dispersion of the simple. Y-axis reported as log2 scale. Follow-up reported in months, age in years old. Duration time of surgery in minutes. Resection length in cm.

## Data Availability

The datasets used and/or analyzed during the current study are available from the corresponding author on reasonable request.

## Abbreviations

PFR: proximal femur replacements
MSTS: Muscoloskeletal Tumor Society scoring system
