# Supplementary figures and images for: Cemented vs uncemented megaprostheses in proximal femur metastases: a multicentric comparative study
Source: BMC Musculoskelet Disord. 2022 Sep 6;22(Suppl 2):1068. doi: 10.1186/s12891-022-05726-7 (PMC9450228; doi:10.1186/s12891-022-05726-7)

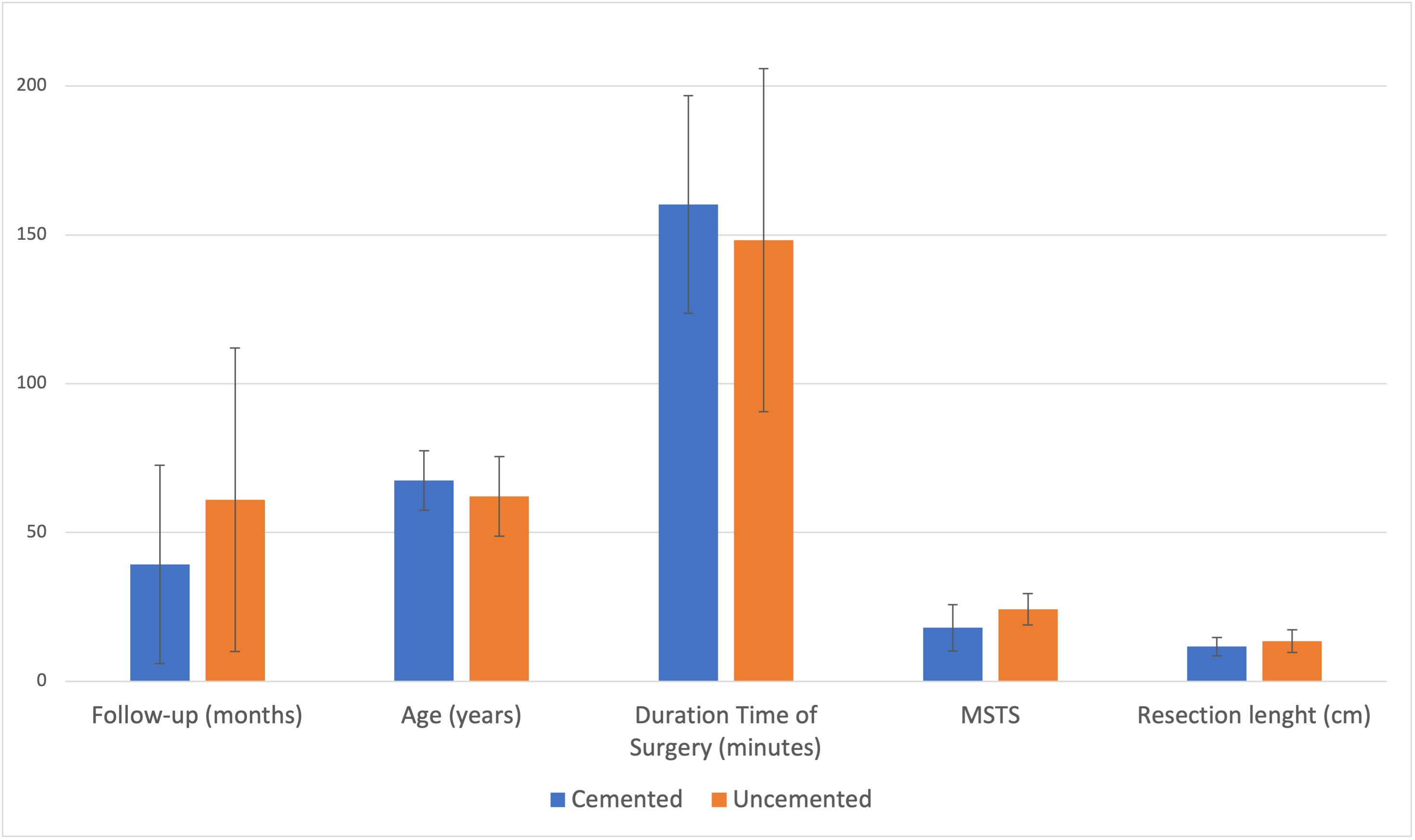

Supplement: Supplementary file 1 — Additional file 1: Supplementary Fig. 1. Supplementary data reported with confidence interval. Follow-up reported in months, age in years old. Duration time of surgery in minutes. Resection length in cm. * for statistical significance of p = 0.001. [file 12891_2022_5726_MOESM1_ESM.tiff]

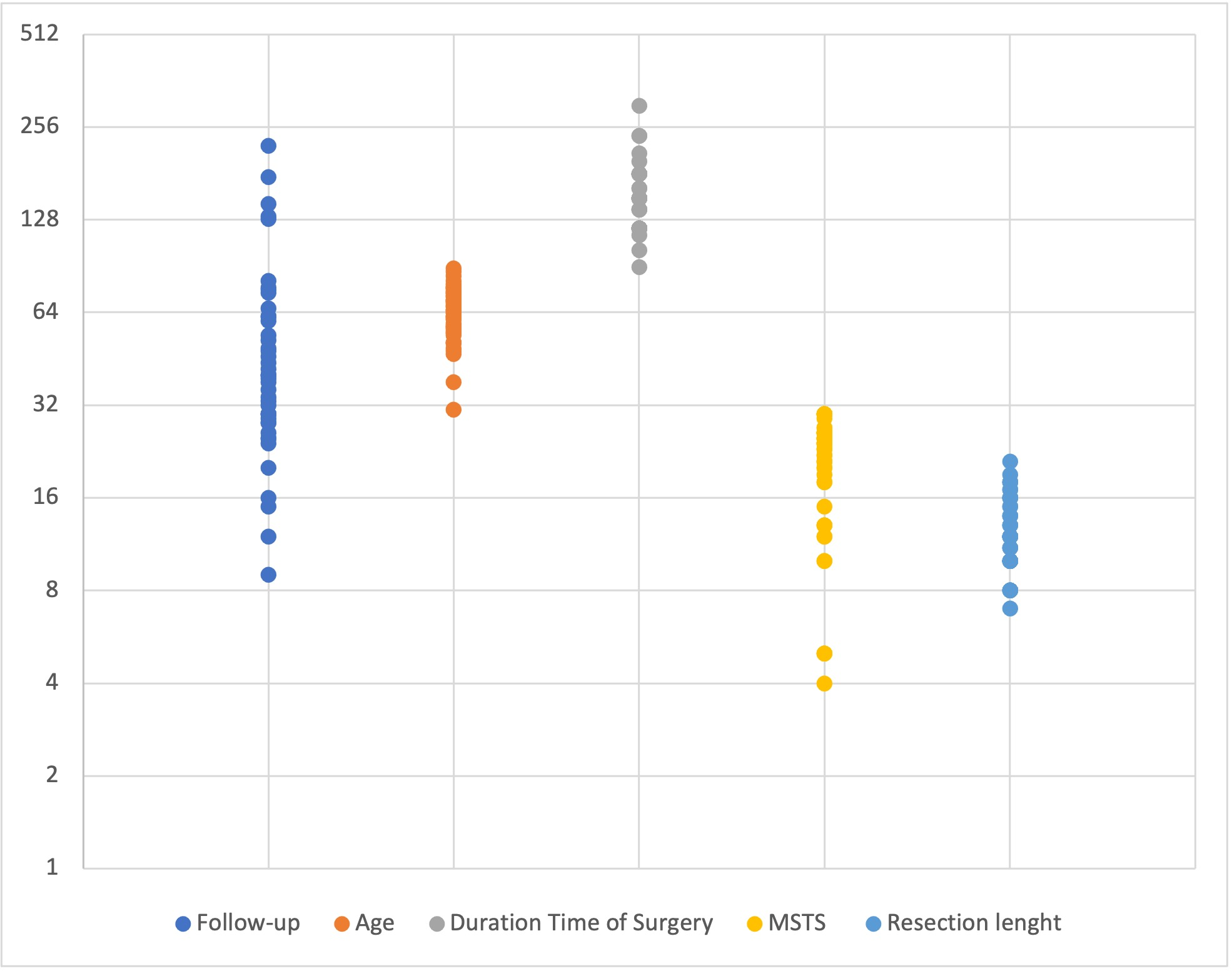

Supplement: Supplementary file 2 — Additional file 2: Supplementary Fig. 2. Dispersion of the simple. Y-axis reported as log2 scale. Follow-up reported in months, age in years old. Duration time of surgery in minutes. Resection length in cm. [file 12891_2022_5726_MOESM2_ESM.tiff]
